# Rising trends on folic acid test requests in a middle-income large academic hospital: A low-value care target for improvement

**DOI:** 10.1016/j.clinsp.2025.100707

**Published:** 2025-06-26

**Authors:** Bruno Adler Maccagnan Pinheiro Besen, Fabio Augusto Rodrigues Gonçalves, João Vitor Ziroldo Lopes, Evelinda Marramom Trindade, Guilherme Henrique Hencklain Fonseca, Luiz Augusto Marcondes Fonseca, Nairo Massakazu Sumita, Arnaldo Lichtenstein, Leila Antonangelo

**Affiliations:** a Medical Sciences Postgraduate Programme, Departamento de Clínica Médica, Faculdade de Medicina, Universidade de São Paulo (FMUSP), São Paulo, SP, Brazil; b Medical ICU, Hospital das Clínicas, Faculdade de Medicina, Universidade de São Paulo (HCFMUSP), São Paulo, SP, Brazil; c Intensive Care Unit, A.C. Camargo Cancer Center, São Paulo, SP, Brazil; d LIM-03 and Clinical Laboratory, Hospital das Clínicas, Faculdade de Medicina, Universidade de São Paulo (HCFMUSP), São Paulo, SP, Brazil; e Faculdade de Medicina, Universidade de São Paulo (FMUSP), São Paulo, SP, Brazil; f Health Technology Assessment hub, Hospital das Clínicas, Faculdade de Medicina, Universidade de São Paulo (HCFMUSP), São Paulo, SP, Brazil; g Hematology Division, Internal Medicine Department, Hospital das Clínicas, Faculdade de Medicina, Universidade de São Paulo (HCFMUSP), São Paulo, SP, Brazil; h Allergy and Immunology Division, Internal Medicine Department, Hospital das Clínicas, Faculdade de Medicina, Universidade de São Paulo (HCFMUSP), São Paulo, SP, Brazil; i General Internal Medicine Division, Department of Internal Medicine, Hospital das Clínicas, Faculdade de Medicina, Universidade de São Paulo (HCFMUSP), São Paulo, SP, Brazil

**Keywords:** Quality, Serum folate, Clinical pathology, Rational use, Low-value care

## Abstract

**Objectives:**

To evaluate the trends in serum folate test ordering and their abnormal results.

**Methods:**

The authors retrieved data from an anonymized laboratory database from a single, large, academic hospital from Brazil. The authors retrieved serum folate test results, the number of repetitions and other laboratory measurements obtained in the same time window, ordering setting and clinic. The authors then evaluated the trends in test ordering, and prevalence of low (< 3 ng/dL) results, and assessed targets for improvement in test ordering.

**Results:**

From January 1st, 2018, to December 31st, 2022, 181,379 folate tests were ordered from 82,052 patients, with 0.7 % of low results, rising from 33,988 in 2018 to 44,187 in 2022. The outpatient setting was the most frequent (89.5 %). The clinics that most frequently ordered folate tests were Gastroenterology (33 %) and Nephrology (9.8 %). Most patients had more than one test ordered (74.8 %). The 135,787 re-tests were performed in 36,300 patients. Many exams were repeated within three (22,570) or six months (46,881). Only 24,243 repetitions were made more than a year after the prior exam.

**Conclusions:**

Folate test ordering is an increasing trend in this large academic hospital, suggestive of non-clinical guided test ordering and low-value testing.

## Highlights

• Folate test ordering is rising in this large academic hospital.
• Only 0.7 % of folate tests have results in the low range (< 3 ng/mL).
• Most patients had more than one test ordered (74.8 %), many within three months.
• Non-clinical guided test ordering suggests low-value testing of folate.
• Almost $600 K dollars were spent on low-value folate ordering over 5-years.

## Introduction

Rational use of health care resources is of utmost importance, mainly in developing countries, to ensure the sustainability of the health care system. Laboratory tests are valuable tools for improving diagnostic accuracy. Ensuring that clinical laboratory tests are properly used should be an integral part of sound healthcare system management. In a clinical laboratory of a public tertiary care university hospital, the portfolio of tests available is extensive, making it easier to order laboratory tests, some of which are unnecessary for diagnosis or patient follow-up.^1^ However, laboratory tests should be used to answer a specific question as inappropriate ordering will lead to many false positives and misdirected investigations. In addition, this increases costs and overburdens nursing and laboratory teams.

Folate is a water-soluble vitamin (B9) that participates as a coenzyme in single-carbon transfers in amino acids metabolism and nucleic acids synthesis - an important factor in the synthesis and repair of DNA and amino acids, as well as in the methylation process, with an essential role in cell division.^2^ Folate is not synthesized by humans requiring that it be obtained from exogenous sources.^3^ An insufficient diet intake, deficient absorption, increase in demand or increased excretion are some causes of its deficiency.^4^ Insufficient intake may be related to overcooking of fruit and vegetables, alcoholism, and parenteral nutrition. Deficient absorption includes congenital causes, celiac disease, inflammatory bowel disease, or intestinal bacterial overgrowth. Inappropriate use of folate may be due to congenital causes, alcoholism, or drugs, such as methotrexate (Methotrexate), metformin (Glucophage), cholestyramine (Prevalite Powder), oral contraceptives, antacids, alcohol, or anticonvulsants, as well as antagonistic drugs like 5-fluorouracil, hydroxyurea, pyrimethamine, trimethoprim/sulfamethoxazole, pentamidine, anti-inflammatory drugs or pancreatic enzymes. An increase in demand can be due to pregnancy, lactation or infancy, and hemolysis. Finally, increased excretion is associated with peritoneal dialysis or hemodialysis.

A sufficient nutritional intake is essential to avoid deficiency and its associated diseases – mainly fetal neural tube defects and megaloblastic anemia.^5^ Other possible undesired associations include cancer.^4^ The supplementation of wheat flour with folic acid – a synthetic oxidized form of folate ‒ started in the United States and Canada in 1998, followed by Chile in 2000, Brazil in 2002 and Argentina in 2003 in South America. With the implementation of such policies, the incidence of both folate deficiency and neural tube defects dropped significantly.^6^

As a result, serum folate testing has now limited indications, as current scientific consensus recommends supplementation therapy as more efficient than folate determination, in the setting of known or presumed folate deficiency.^7^ However, the medical literature recurrently reports a high number of folate requests, from different centers and countries.^8^, ^9^, ^10^

At the present study’s center, the authors have observed an increase in the ordering of serum folate, raising concerns about its clinical need and the associated unnecessary rise in costs. In the present study, the authors aimed to explore the trends and the value of ordering serum folate results from the clinical laboratory of the present study’s hospital over a period of five years.

## Material and methods

### Study design

This is an observational study undertaken at Instituto Central, Hospital das Clinicas, Faculdade de Medicina, Universidade de São Paulo (HCFMUSP), SP, Brazil. The HCFMUSP is a tertiary, university-based medical facility. The study protocol was approved by the HCFMUSP Ethics Committee (CAPPesq 70,283,017.7.0000.0068) with a waiver of informed consent due to the use of anonymized retrospective data. The authors report these results in accordance with STROBE guidance.^11^

### Data collection

The authors collected data on all serum folate determinations requested for both inpatients and outpatients, from January 01, 2018, to December 31, 2022. The authors then collected data from the complete blood count, Lactate Dehydrogenase (LDH), haptoglobin and bilirubin in a one-week window (3 days before up to 3 days later) of the folate measurement. The authors also retrieved patients’ age, sex, the ordering medical specialty and the setting where the test was ordered (outpatient, inpatient, intensive care unit, emergency department, day hospital).

### Measurements

The Complete Blood Cell Count (CBC) with differential was carried out on the SYSMEX XN-9100 hematology analyzer and Sysmex DI-60 automated digital cell morphology system (Sysmex Corporation, Kobe, Japan). The folic acid (folate) levels were analyzed on a Cobas e602 analyzer (Roche Diagnostics, Basel, Switzerland) using electrochemiluminescence immunoassay. The total and direct bilirubin levels were analyzed on a Cobas c702 analyzer (Roche Diagnostics, Basel, Switzerland) using the colorimetric method. The indirect bilirubin level was calculated by subtracting the direct value from the total value. The lactate dehydrogenase activity was analyzed on a Cobas c702 analyzer (Roche Diagnostics, Basel, Switzerland) using the ultraviolet method. The haptoglobin levels were analyzed on a Cobas c502 analyzer (Roche Diagnostics, Basel, Switzerland) using an immunoturbidimetric assay. Reference values for all measurements are presented in the Supplemental File.

### Analytical framework

The authors first assessed the temporal trends in serum folate test measurements through the 5-year timeframe. Then, the authors determined the prevalence of low folate measurements. An acid folic level of ≤ 2.99 ng/mL was considered “low”, while measurements ≥3 ng/mL were considered non-low.^12^ Finally, to assess whether folate measurements were ordered in the context of a hematological manifestation, the authors stratified the tests into three non-exclusive subgroups according to: 1) Possible hemolysis (increased unconjugated bilirubin and increased LDH and low haptoglobin); 2) Macrocytosis (Mean Corpuscular Volume [MCV] levels > 100 fL) and 3) Pancytopenia (anemia AND leukopenia AND thrombocytopenia). The authors used reference cut-offs to classify these results (Supplemental File). The concurrent occurrence of these hematological abnormalities with folate test ordering was then examined.

### Statistical analysis

In the descriptive analysis, the authors present categorical variables as counts and proportions; and continuous variables, as medians and interquartile ranges. For comparisons, the authors used the Chi-Square test or the Wilcoxon Rank Sum Test (or the Kruskall-Wallis test) for categorical and continuous variables, respectively. The authors performed a separate analysis for the pediatric population. All statistical tests were two-sided. The authors present the time series as line plots with a linear fit. Bar graphs, violin plots, and pie charts are presented for descriptive purposes as appropriate. Statistical analyses were performed using R (v4.1.0) and Stata SE 18.0. A p-value < 0.05 was considered statistically significant.

## Results

### Temporal trends in folate ordering

**Fig. 1:**
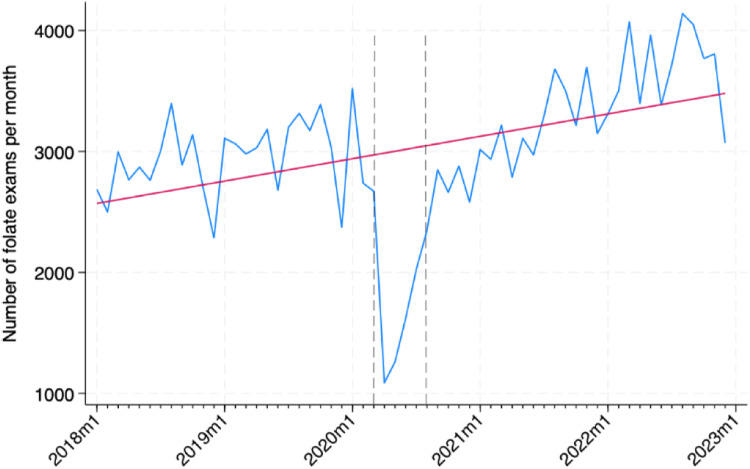
Temporal trend of the number of folate exams/month. The blue line depicts the number of folate exams per month, while the red line describes the linear trend. The darker dashed gray lines depict the 1st wave of the pandemic.

From January 01, 2018, to December 31, 2022, 181,379 folate tests were processed by the central laboratory division, from 82,052 patients. Fig. 1 presents the increasing trend of folate tests observed during the study period, regardless of the 1st wave of the pandemic.

**Fig. 2:**
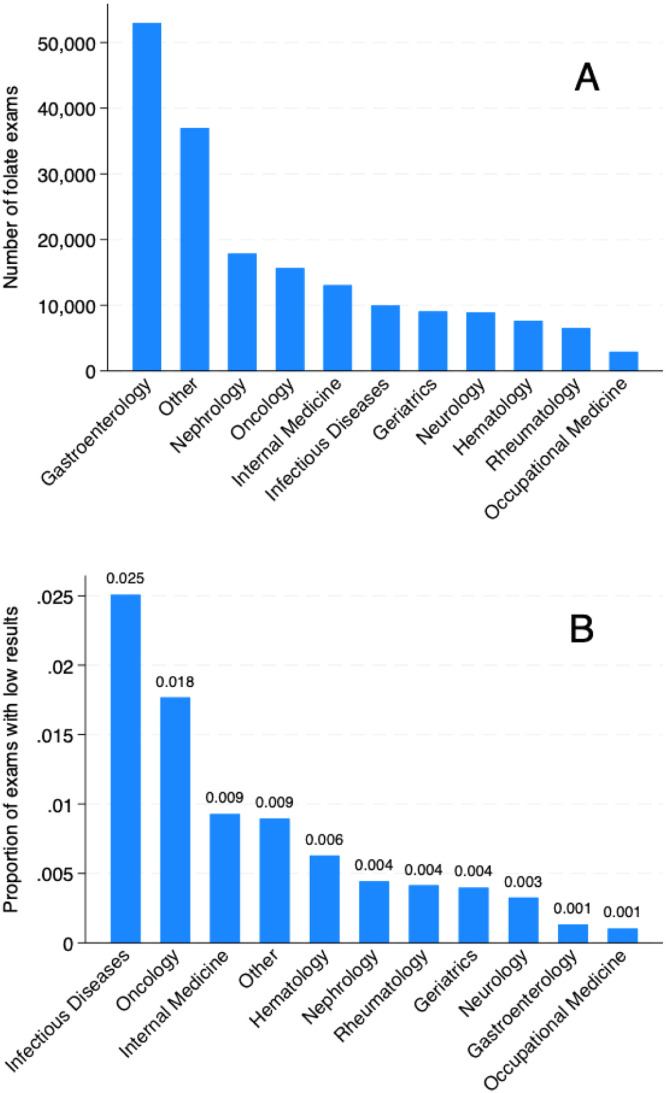
Bar graph of the most common ordering clinics of folate exams and their proportion of exams with low results.

Table 1 presents a yearly variation in folate test ordering, along with some patient and ordering clinic and setting characteristics. Overall, the median age and female sex proportion, although statistically different, did not differ to a clinically relevant extent. Median folate levels ranged from 10.5 to 11.6, while the proportion of patients with low folate levels ranged from 0.5 % to 0.9 %. The most common scenario of ordering folate was in the outpatient scenario, ranging from 88.7 % to 90.6 %. The clinics that most frequently ordered folate tests were respectively Gastroenterology, Nephrology, Oncology, Internal Medicine, and Infectious Diseases, as presented in Fig. 2A. However, the proportion of altered exams was different between clinics (Fig. 2B). Table S1 presents these results for the pediatric population, in which more ordering in the inpatient setting (approximately 15 %) was observed.

**Table 1:** Serum folate ordering characteristics from 2018 to 2022.

Variable	2018	2019	2020	2021	2022	p-value
N	33,988	36,531	28,249	38,584	44,187	
Age, median (IQR)	55.0 (40.0, 68.0)	56.0 (41.0, 68.0)	55.0 (40.0, 67.0)	56.0 (41.0, 68.0)	56.0 (41.0, 68.0)	<0.001
Female, n ( %)	21,234 (62.5 %)	22,705 (62.2 %)	17,293 (61.2 %)	23,557 (61.1 %)	27,008 (61.1 %)	<0.001
Folate (ng/mL), median (IQR)	10.9 (7.6, 15.1)	11.6 (8.2, 15.8)	11.1 (7.7, 15.0)	10.9 (7.5, 14.7)	10.5 (7.4, 14.2)	<0.001
Low folate, n ( %)	244 (0.7 %)	201 (0.6 %)	227 (0.8 %)	361 (0.9 %)	238 (0.5 %)	<0.001
Clinic						<0.001
*Gastroenterology*	10,655 (31.3 %)	11,288 (30.9 %)	8661 (30.7 %)	11,102 (28.8 %)	11,270 (25.5 %)	
*Nephrology*	2613 (7.7 %)	3492 (9.6 %)	2898 (10.3 %)	4227 (11.0 %)	4625 (10.5 %)	
*Internal Medicine*	2756 (8.1 %)	2746 (7.5 %)	1915 (6.8 %)	2525 (6.5 %)	3108 (7.0 %)	
*Oncology*	2483 (7.3 %)	2883 (7.9 %)	2690 (9.5 %)	3316 (8.6 %)	4294 (9.7 %)	
*Neurology*	1463 (4.3 %)	1772 (4.9 %)	1273 (4.5 %)	1844 (4.8 %)	2547 (5.8 %)	
*Geriatrics*	2086 (6.1 %)	2070 (5.7 %)	1236 (4.4 %)	1734 (4.5 %)	1929 (4.4 %)	
*Hematology*	1563 (4.6 %)	1527 (4.2 %)	1011 (3.6 %)	1614 (4.2 %)	1915 (4.3 %)	
*Rheumatology*	1103 (3.2 %)	1123 (3.1 %)	1013 (3.6 %)	1479 (3.8 %)	1796 (4.1 %)	
*ID*	1691 (5.0 %)	1620 (4.4 %)	1716 (6.1 %)	2308 (6.0 %)	2637 (6.0 %)	
*OM*	494 (1.5 %)	801 (2.2 %)	263 (0.9 %)	452 (1.2 %)	908 (2.1 %)	
*Other*^a^	7081 (20.8 %)	7209 (19.7 %)	5573 (19.7 %)	7983 (20.7 %)	9158 (20.7 %)	
Setting						<0.001
*ICU*	234 (0.7 %)	290 (0.8 %)	390 (1.4 %)	452 (1.2 %)	425 (1.0 %)	
*Inpatient*	2629 (7.7 %)	2980 (8.2 %)	2409 (8.5 %)	3272 (8.5 %)	3516 (8.0 %)	
*Outpatient*	30,778 (90.6 %)	32,754 (89.7 %)	25,068 (88.7 %)	34,281 (88.8 %)	39,558 (89.5 %)	
*ED*	139 (0.4 %)	297 (0.8 %)	205 (0.7 %)	335 (0.9 %)	362 (0.8 %)	
*Day hospital*	208 (0.6 %)	210 (0.6 %)	177 (0.6 %)	244 (0.6 %)	326 (0.7 %)	
Repetitions						<0.001
*None*	9508 (28.0 %)	8949 (24.5 %)	6424 (22.7 %)	8706 (22.6 %)	12,165 (27.5 %)	
*At least one*	24,480 (72.0 %)	27,582 (75.5 %)	21,825 (77.3 %)	29,878 (77.4 %)	32,022 (72.5 %)	
Number of exams per patient						<0.001
*1*	9508 (28.0 %)	8949 (24.5 %)	6424 (22.7 %)	8706 (22.6 %)	12,165 (27.5 %)	
*2*	5675 (16.7 %)	6195 (17.0 %)	4369 (15.5 %)	6455 (16.7 %)	8088 (18.3 %)	
*3*	4070 (12.0 %)	4553 (12.5 %)	3315 (11.7 %)	4842 (12.5 %)	5459 (12.4 %)	
*4*	3193 (9.4 %)	3680 (10.1 %)	2853 (10.1 %)	4035 (10.5 %)	4239 (9.6 %)	
*5*	2667 (7.8 %)	2978 (8.2 %)	2443 (8.6 %)	3338 (8.7 %)	3414 (7.7 %)	
*6*	2068 (6.1 %)	2415 (6.6 %)	1999 (7.1 %)	2543 (6.6 %)	2609 (5.9 %)	
*7*	1651 (4.9 %)	1908 (5.2 %)	1624 (5.7 %)	2006 (5.2 %)	1967 (4.5 %)	
*8*	1237 (3.6 %)	1467 (4.0 %)	1255 (4.4 %)	1577 (4.1 %)	1512 (3.4 %)	
*9*	945 (2.8 %)	1038 (2.8 %)	929 (3.3 %)	1174 (3.0 %)	1089 (2.5 %)	
*10+*	2974 (8.8 %)	3348 (9.2 %)	3038 (10.8 %)	3908 (10.1 %)	3645 (8.2 %)	

IQR, Interquartile Range; ID, Infectious Diseases; OM, Occupational Medicine; ICU, Intensive Care Unit; ED, Emergency Department.

a Other clinics include other medical, surgical, obstetrics, psychiatry and other multidisciplinary clinics.

**Fig. 3:**
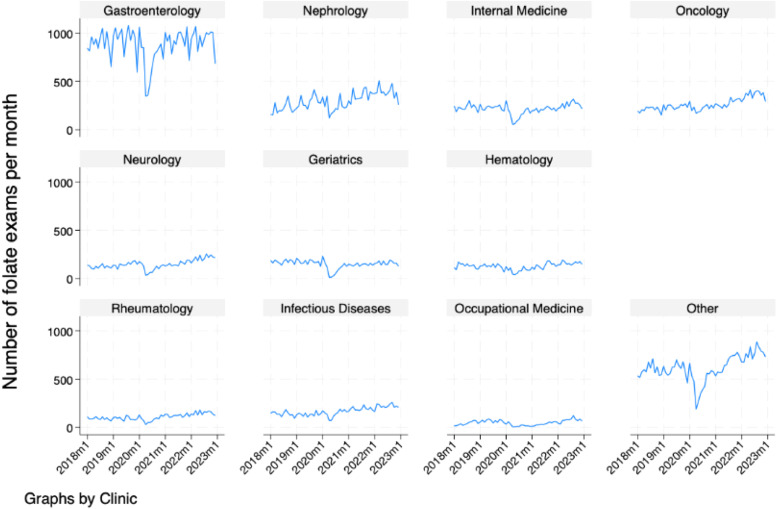
Temporal trend of the number of folate exams/month stratified by ordering clinic.

Fig. 3 demonstrates the increasing monthly trend of folate test ordering by each of the 10 most common ordering clinics. By visual inspection, the authors observe that most clinics follow the same overall trend of the time series, except for internal medicine, gastroenterology and geriatrics, whose series seem stationary.

### Serum folate results

**Fig. 4:**
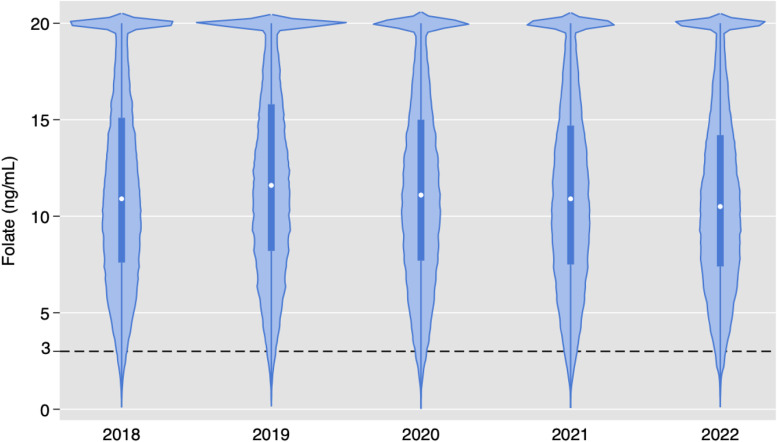
Violin plot for serum folate test results. The dashed line depicts the reference value for low values of folate (≤2.99 ng/mL).

Of the 181,539 folate orders, only 1272 (0.7 %) had low levels. Fig. 4 demonstrates yearly violin plots for serum folate test results. Although some variation is observed, there is no trend in results. Figure S1 demonstrates violin plots in the pediatric population, with no major difference in results compared to adults.

Table 2 presents the proportion of low serum folate results for each of the studied characteristics. A small difference in patients’ age and sex was associated with low serum folate results, while laboratory characteristics associated with hematological manifestations presented an increased proportion of low levels, especially for pancytopenia (1.89 %). Regarding the setting, the outpatient setting was associated with a very low probability of low results (0.36 %) compared to ICU (7.43 %) and inpatient (3.54 %) settings. Among the most commonly ordered clinics, infectious diseases, (2.51 %) and oncology (1.77 %) had a higher probability of low levels. Finally, repeated exams were more likely to be normal (0.61 %) compared to non-repeated exams (0.97 %). Table S2 presents these results for the pediatric population (0.56 %). The only variable associated with low levels of folate was the setting of ordering.

**Table 2:** Proportion of low levels of serum folate test results according to the studied characteristics.

Variable	Total	Low	Not low	p-value
N	181,539	1271 (0.70 %)	180,268 (99.30 %)	
Age, median (IQR)		52 (37, 65)	56 (41, 68)	<0.001
Sex				<0.001
*Male*	69,742	671 (0.96 %)	69,071 (99.04 %)	
*Female*	111,797	600 (0.54 %)	111,197 (99.46 %)	
Year				<0.001
*2018*	33,988	244 (0.72 %)	33,744 (99.28 %)	
*2019*	36,531	201 (0,55 %)	36,330 (99.45 %)	
*2020*	28,249	227 (0.80 %)	28,022 (99.20 %)	
*2021*	38,584	361 (0.94 %)	38,223 (99.06 %)	
*2022*	44,187	238 (0.54 %)	43,949 (99.46 %)	
Clinic				<0.001
*Gastroenterology*	59,976	70 (0.13 %)	52,906 (99.87 %)	
*Nephrology*	17,855	79 (0.44 %)	17,776 (99.56 %)	
*Internal Medicine*	13,050	121 (0.93 %)	12,929 (99.07 %)	
*Oncology*	15,666	277 (1.77 %)	15,389 (98.23 %)	
*Neurology*	8899	29 (0.33 %)	8870 (99.67 %)	
*Geriatrics*	9055	36 (0.40 %)	9019 (99.60 %)	
*Hematology*	7630	48 (0.63 %)	7582 (99.37 %)	
*Rheumatology*	6514	27 (0.41 %)	6487 (99.59 %)	
*ID*	9972	250 (2.51 %)	9722 (97.49 %)	
*OM*	2918	3 (0.10 %)	2915 (99.90 %)	
*Other*	37,004	331 (0.89 %)	36,673 (99.11 %)	
Setting				<0.001
*ICU*	1791	133 (7.43 %)	1658 (92.57 %)	
*Inpatient*	14,806	524 (3.54 %)	14,282 (96.46 %)	
*Outpatient*	162,439	577 (0.36 %)	161,862 (99.64 %)	
*ED*	1338	30 (2.24 %)	1308 (97.76 %)	
*Day hospital*	1165	7 (0.60 %)	1158 (99.40 %)	
Repetitions				<0.001
*None*	45,752	444 (0.97 %)	45,308 (99.03 %)	
*At least one*	135,787	827 (0.61 %)	134,960 (99.39 %)	
Clinical characteristics				
*Hemolysis*	355 (0.20 %)	5 (1.41 %)	350 (98.59 %)	0.108
*Pancytopenia*	1745 (0.96 %)	33 (1.89 %)	1712 (98.11 %)	<0.001
*Macrocytosis*	21,709 (11.96 %)	211 (0.98 %)	21,498 (99.02 %)	<0.001

IQR, Interquartile Range; ID, Infectious Diseases; OM, Occupational Medicine; ICU, Intensive Care Unit; ED, Emergency Department.

### Repetitions

**Fig. 5:**
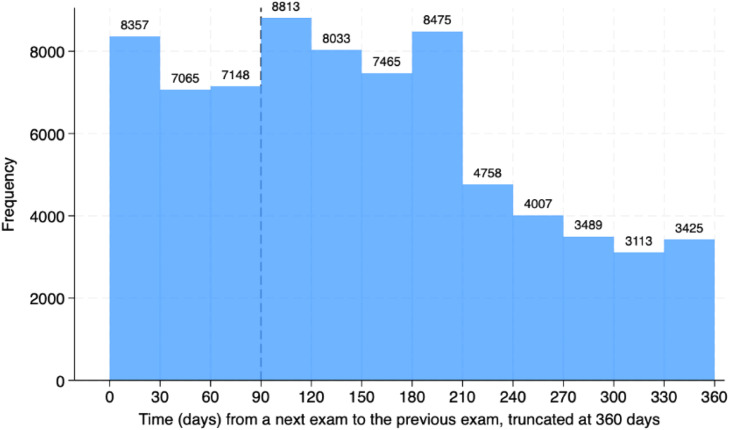
Histogram of the timing of serum folate exams repetitions. The dashed line depicts 90 days from a first test to a repeated test.

**Fig. 6:**
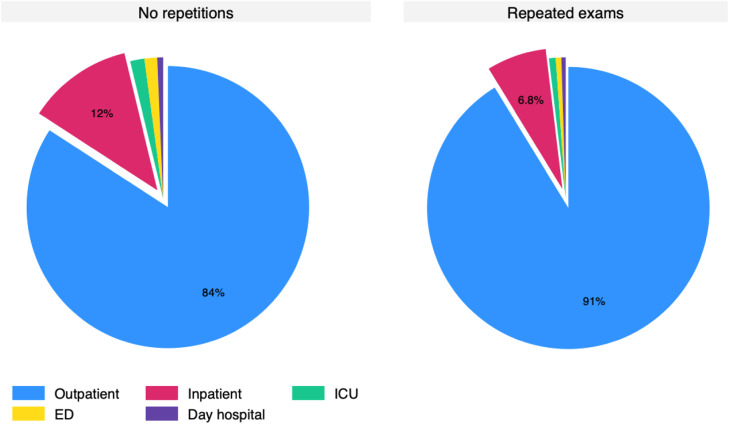
Order setting of exams with repetitions and without repetitions. ICU, Intensive Care Unit; ED, Emergency Department; inpatient refers to patients admitted to hospital, but not on ED or ICUs.

Most patients had more than one test ordered (72 % to 77.4 %) and roughly 10 % of orders were made 10 or more times in the same patient. The 135,787 re-tests were performed in 36,300 patients. Amid the re-tests, there was only 0.61 % (827 tests) resulting in low (≤ 2.99 ng/mL). The histogram for the time since a previous exam is presented in Fig. 5, which demonstrates that many exams were repeated within three (22,570) or six months (46,881). Only 24,243 repetitions were made more than a year after the prior exam. Pediatric data mirrored adult trends, with 0.56 % low folate rates and frequent repetitions (Fig. S2). The distribution of non-repeated and repeated tests according to setting is presented in Fig. 6 and Figure S3 for the pediatric population. The outpatient setting was even more frequent among repeated exams.

## Discussion

### Statement of main findings

In the current study, the authors observed that only 0.7 % of the 180,319 folate tests performed in the hospital over a five-year timeframe presented with low results, despite a high financial cost and likely little clinical benefit. Interestingly, serum folate test ordering is an increasing trend, regardless of current recommendations to avoid ordering the test routinely in countries with fortification of flour. The tests were more frequently ordered in the outpatient setting and a few clinics were responsible for most of the tests ordered. The authors also observed a pattern of repetition of exams that suggests a non-clinical guided test ordering, as we have previously observed in the inpatient setting for a wider range of laboratory tests.^13^

Similar results have been reported by others. In the Beth Israel Deaconess Medical Center, for example, outpatient serum folate results were deemed low in 0.06 % of tests in an 11-year time span and the authors concluded there was a substantial overuse of the test in their center.^14^ Canadian studies pointed out that in 1994–95, before food supplementation with folic acid, 7,92 million dollars were spent on folate tests.^15^ More recently, a study showed that less than one percent of the tests revealed folate deficiency and that 32 thousand dollars could have been saved in a single hospital if the tests were restricted.^16^ In the hospital, the costs of a single serum folate test are $3.32 dollars per exam, which would correspond to a cost of $598,659.08 dollars in the analyzed period.

The lower reference range of laboratory tests is usually set to encompass the lower 2.5 % of the population. Therefore, by random chance, one would expect 2.5 % of any population to have values below this reference range. In the present results, only 0.7 % of the study population has such values, which suggests that with the advent of folate supplementation, the population values of folate have shifted. The test therefore becomes irrelevant in most of the sampled population and folate deficiency is extremely rare. In fact, when the pre-test probability is this low, a false positive becomes more likely as the true positive rate declines. This makes a strong case for ordering the test only in selected cases with a clinical picture of folate deficiency.

In Brazil, a study conducted in the city of Sao Paulo showed that only 1.78 % of the population had serum levels compatible with folate deficiency.^6^ In the landmark paper published by the Centers for Disease Control and Prevention, the authors observed a 0.3 % proportion of low serum folate after folic acid fortification.^17^ Finally, even in the pediatric population, in countries without mandated flour fortification, the clinical value of folate test order is questioned. In a study in Israel, of >20,000 children who tested for serum folate, only 4.3 % had folic acid deficiency, of which only two patients (0.2 %) had megaloblastic anemia, which also suggests non-clinical test ordering.^18^ In the present study, the authors observed a similar pattern to the adult population, with a 0.56 % prevalence of low levels.

Since routine fortification of food with folic acid has become a public policy, and considering the difficulty in the diagnosis of folate deficiency based on usual serum results,^19^ many stakeholders have strived to reduce testing of serum folate in conditions at increased risk of deficiency.^9^ Whether folate testing should be done in this scenario is likely not recommended in countries like Brazil. Hence, one would expect a decreasing trend in its ordering, unlike the present results, with an increasing and unexplained trend in folate test ordering. Nevertheless, ordering serum folate may be considered in a few circumstances where serum folate deficiency could be part of the clinical picture to avoid missed diagnoses.

Algorithms recommend that in the presence of anemia with elevated MCV and non-elevated reticulocytes, folic acid should be measured, in addition to vitamin B12, homocysteine and methylmalonic acid, besides examining the peripheral smear.^20^^,^^21^ Unfortunately, this suggested algorithm for investigation is not followed, as shown in a Korean study.^22^ This may lead to underutilization and improper diagnoses, either due to low availability and lack of knowledge or because of overreliance on folate test ordering. The pattern of repetitions in this study, however, suggests an automated ordering of the test with no clinical reasoning based on clinical hypotheses. In the present study, the authors explored ordering due to hematological laboratory alterations that could prompt clinicians to order the exam. Interestingly, Hematology was not one of the primary ordering clinics and the authors did not observe a higher serum folate deficiency proportion in patients with likely hemolysis, pancytopenia, or macrocytosis.

The present results suggest that the outpatient setting is the main target for improvement of low-value use of folate tests, especially in the Gastroenterology and Nephrology clinics, which represent more than a third of tests ordered in this time span. Although malabsorptive states in gastroenterology and increased elimination in patients undergoing dialysis may reduce serum folate levels, testing should likely not be routinely done in these scenarios, due to the low prevalence of folate deficiency. Testing should be considered when a clinical indication, as the authors described above, presents.

Different centers have tried a variety of strategies to reduce folate tests in clinical practice: University Health Network restricted RBC folate requests to hematologists and gastroenterologists, removing access for other clinicians. This strategy reduced RBC folate requests by 94 %.^23^ The London Health Sciences Centre (LHSC)/St. Joseph’s Healthcare London (SJHC) implemented targeting e-mails, educational memos, decision support pop-ups in the exam requesting system as well as a requirement for biochemist approval, which ultimately resulted in a reduction of folate tests by 98 %.^24^ At Johns Hopkins University School of Medicine, a nonintrusive strategy with appropriate indications in text that accompanied the laboratory orders was incorporated. In this study, folate orders already were on a pre-existing decrease, which reduced the impact of the intervention.^25^ In the hospital, a clinical decision algorithm improved the appropriateness of calcium test ordering, which may still be a possible policy measure for improvement.^26^ In the hospital and others with increasing trends, as the availability of laboratory exams may trigger the demand,^27^ policy measures to avoid its non-clinical ordering are necessary to curb its low-value utilization in practice. Educational interventions or policy measures could simultaneously reduce overuse in outpatients and improve targeted testing for macrocytic anemia.

The present study’s main strength is the large volume of the center, which provides a large sample size to understand potential targets for improvement. However, the present study has some limitations. First, as the authors only had access to laboratory data, information was missing for patients’ clinical characteristics that may have motivated clinicians to order the test. Second, the authors had no data on the prescription of oral folate for replenishment. Nevertheless, the demonstrated ordering patterns are still compatible with the non-clinically oriented ordering of the exam, with substantial room for improvement to tackle low-value care. Third, the present study is a single-center study from a large academic health center, that may not be representative of other centers in Brazil or other low- or middle-income countries, but these results are still relevant as a potential target to address low-value care.

## Conclusion

The prevalence of folate deficiency was very low in the high-complexity hospital, likely due to the consumption of folic acid-enriched wheat flour, which has been mandatory in Brazil since 2002. Despite current recommendations to avoid folate test ordering, the authors observed an increasing trend in ordering the exam, suggestive of non-clinically guided ordering. Policy measures, such as the implementation of electronic health record alerts for folate test ordering in specific scenarios, are needed to reduce low-value test ordering. Clinicians should be aware of the very low yield of these tests, especially in the outpatient setting, and refrain from ordering it except when clinically recommended.

## CRediT authorship contribution statement

**Bruno Adler Maccagnan Pinheiro Besen:** Conceptualization, Formal analysis, Writing – original draft, Writing – review & editing, Visualization. **Fabio Augusto Rodrigues Gonçalves:** Formal analysis, Writing – original draft, Writing – review & editing, Data curation, Visualization. **João Vitor Ziroldo Lopes:** Writing – review & editing. **Evelinda Marramom Trindade:** Conceptualization, Writing – review & editing, Supervision. **Guilherme Henrique Hencklain Fonseca:** Writing – review & editing. **Luiz Augusto Marcondes Fonseca:** Writing – review & editing. **Nairo Massakazu Sumita:** Resources, Writing – review & editing. **Arnaldo Lichtenstein:** Conceptualization, Writing – original draft, Writing – review & editing, Supervision. **Leila Antonangelo:** Conceptualization, Resources, Writing – review & editing, Supervision.

## Conflicts of interest

The authors declare no conflicts of interest.

## Appendix Supplementary materials

Image, application 1
