# Supplementary material for: Rising trends on folic acid test requests in a middle-income large academic hospital: A low-value care target for improvement
Source: Clinics (Sao Paulo). 2025 Jun 26;80:100707. doi: 10.1016/j.clinsp.2025.100707 (PMC12241388; doi:10.1016/j.clinsp.2025.100707)
Supplement: Supplementary file 1 [file mmc1.docx]

**CLINICS-D-24-00611_Supplementary Materials**

**Rising trends on folic acid test requests in a middle-income large academic hospital: a target to reduce low value care**

**Supplemental File**

Sumário

[Supplemental Methods 2](#_Toc194049684)

[Supplemental results 4](#_Toc194049685)

[**Table S1:** Serum folate ordering characteristics from 2018 to 2022 in the pediatric population 4](#_Toc194049686)

[**Table S2:** Proportion of low levels of serum folate test results according to the studied characteristics in the pediatric population 5](#_Toc194049687)

[**Figure S1:** Violin plot for serum folate test results in the pediatric population 6](#_Toc194049688)

[**Figure S2:** Histogram of the timing of serum folate exams repetitions in the pediatric population 7](#_Toc194049689)

[**Figure S3:** Order setting of exams with repetitions and without repetitions in the pediatric population 8](#_Toc194049690)

## Supplemental Methods

Reference values used for the purposes of this analysis were the following:

**Unconjugated bilirrubin:** < 0.7 mg/dL

**Lactic Desidrogenase (LDH)**

Women : 135-214 U/L

Men  : 135-225 U/L

Children ( 2 - 15 years-old ) : 120-300 U/L

**Haptoglobin**

> 1 year to 12 years:

Men : 3 - 270 mg/dL

Women : 11 - 220 mg/dL

> 12 years to 60 years:

Men: 14 - 258 mg/dL

Women : 35 - 250 mg/dL

> 60 years:

Men : 40 - 268 mg/dL

Women : 63 - 272 mg/dL

**Full blood count - RDW-SD**

Men and Women :  35,1 fL - 43,9 fL

**Full blood count – Hemoglobin**

| **Idade** | **Men** | **Women** |
| --- | --- | --- |
| 0 to 6 days | 15,3  -  21,0 g/dL | 15,3  -  21,0 g/dL |
| 7 to 23 days | 14,2  -  17,2 g/dL | 14,2  -  17,2 g/dL |
| 24 to 29 days | 12,2  - 16,0 g/dL | 12,2  - 16,0 g/dL |
| 1 to 36 months | 10,3   -  13,7 g/dL | 10,3   -  13,7 g/dL |
| 3 to 10 anos | 11,7  -  14,4 g/dL | 11,7  -  14,4 g/dL |
| 11 to 15 anos | 14,4  -  16,6 g/dL | 14,4  -  16,6 g/dL |
| > 16 anos | 13,5 - 17,5 g/dL | 11,5 - 15,5 g/dL |

**Full blood count – Leucocytes**

| **Idade** | **Men e Women** |
| --- | --- |
| 0 to 1 day | 9,0 - 38,0 x 10^3^/mm^3^ |
| 2 to 7 days | 5,0 - 30,0 x 10^3^/mm^3^ |
| 8 to 30 days | 5,0 - 21,0 x 10^3^/mm^3^ |
| 2 to 6 months | 5,0 - 15,0 x 10^3^/mm^3^ |
| 7 to 11 months | 6,0 - 18,0 x 10^3^/mm^3^ |
| 1 to 2 anos | 6,0 - 17,0 x 10^3^/mm^3^ |
| 3 to 4 anos | 5,5 - 15,5 x 10^3^/mm^3^ |
| 5 to 12 anos | 4,5 - 13,0 x 10^3^/mm^3^ |
| 13 to 15 anos | 4,5 a 12,0 x 10^3^/mm^3^ |
| > 16 anos | 4,0 - 11,0 x 10^3^/mm^3^ |

**Full blood count – Platelets**

Men and Women :  150  -  400 x 10^3^ /mm^3^

## Supplemental results

Additional results are here presented for the pediatric population.

### Table S1 Serum folate ordering characteristics from 2018 to 2022 in the pediatric population.

| Variable | **2018** | **2019** | **2020** | **2021** | **2022** | p-value |
| --- | --- | --- | --- | --- | --- | --- |
| N | 1043 | 1206 | 909 | 1176 | 1311 |  |
| Age, median (IQR) | 12.0  (7.0, 15.0) | 12.0  (6.0, 15.0) | 12.0  (6.0, 15.0) | 13.0  (7.0, 15.0) | 11.0  (7.0, 15.0) | 0.003 |
| Female, n (%) | 496 (47.6%) | 590 (48.9%) | 452 (49.7%) | 571 (48.6%) | 613 (46.8%) | 0.66 |
| Folate (ng/mL), median (IQR) | 11.3  (7.5, 15.8) | 12.1  (8.3, 16.6) | 12.6  (8.8, 16.3) | 11.9  (8.4, 16.2) | 11.7  (8.1, 15.5) | <0.001 |
| Low folate, n (%) | 5 (0.5%) | 7 (0.6%) | 6 (0.7%) | 8 (0.7%) | 6 (0.5%) | 0.93 |
| Setting |  |  |  |  |  | 0.013 |
| ICU | 5 (0.5%) | 2 (0.2%) | 8 (0.9%) | 9 (0.8%) | 4 (0.3%) |  |
| Inpatient | 155 (14.9%) | 179 (14.8%) | 156 (17.2%) | 184 (15.6%) | 218 (16.6%) |  |
| Outpatient | 870 (83.4%) | 1015 (84.2%) | 729 (80.2%) | 972 (82.7%) | 1084 (82.7%) |  |
| ED | 1 (0.1%) | 0 (0.0%) | 5 (0.6%) | 4 (0.3%) | 2 (0.2%) |  |
| Day hospital | 12 (1.2%) | 10 (0.8%) | 11 (1.2%) | 7 (0.6%) | 3 (0.2%) |  |
| Repetitions |  |  |  |  |  | <0.001 |
| None | 481 (46.1%) | 551 (45.7%) | 383 (42.1%) | 522 (44.4%) | 708 (54.0%) |  |
| At least one | 562 (53.9%) | 655 (54.3%) | 526 (57.9%) | 654 (55.6%) | 603 (46.0%) |  |
| Number of exams per patient |  |  |  |  |  | <0.001 |
| 1 | 481 (46.1%) | 551 (45.7%) | 383 (42.1%) | 522 (44.4%) | 708 (54.0%) |  |
| 2 | 196 (18.8%) | 216 (17.9%) | 161 (17.7%) | 245 (20.8%) | 213 (16.2%) |  |
| 3 | 103 (9.9%) | 127 (10.5%) | 103 (11.3%) | 103 (8.8%) | 104 (7.9%) |  |
| 4 | 48 (4.6%) | 66 (5.5%) | 54 (5.9%) | 53 (4.5%) | 49 (3.7%) |  |
| 5 | 37 (3.5%) | 48 (4.0%) | 36 (4.0%) | 28 (2.4%) | 37 (2.8%) |  |
| 6 | 29 (2.8%) | 49 (4.1%) | 33 (3.6%) | 25 (2.1%) | 15 (1.1%) |  |
| 7 | 28 (2.7%) | 21 (1.7%) | 18 (2.0%) | 11 (0.9%) | 4 (0.3%) |  |
| 8 | 27 (2.6%) | 34 (2.8%) | 19 (2.1%) | 34 (2.9%) | 20 (1.5%) |  |
| 9 | 7 (0.7%) | 14 (1.2%) | 14 (1.5%) | 9 (0.8%) | 7 (0.5%) |  |
| 10+ | 87 (8.3%) | 80 (6.6%) | 88 (9.7%) | 146 (12.4%) | 154 (11.7%) |  |

IQR, interquartile range; ID, infectious diseases; OM, occupational medicine; ICU, intensive care unit; ED, emergency department.

### Table S2 Proportion of low levels of serum folate test results according to the studied characteristics in the pediatric population.

| **Variable** | Total | Low | Not low | p-value |
| --- | --- | --- | --- | --- |
| N | 5,645 | 32 (0.56%) | 5,613 (99.44%) |  |
| Age, median (IQR) |  | 12 (7, 15) | 12.5 (3, 16) | 0.88 |
| Sex |  |  |  | 0.39 |
| *Male* | 2,722 | 13 (0.48%) | 2,709 (99.52%) |  |
| *Female* | 2,923 | 19 (0.65%) | 2,904 (99.35%) |  |
| Year |  |  |  | 0.93 |
| *2018* | 1,043 | 5 (0.48%) | 1,038 (99.52%) |  |
| *2019* | 1,206 | 7 (0.58%) | 1,199 (99.42%) |  |
| *2020* | 909 | 6 (0.66%) | 903 (99.34%) |  |
| *2021* | 1,176 | 8 (0.68%) | 1,168 (99.32%) |  |
| *2022* | 1,311 | 6 (0.46%) | 1,305 (99.54%) |  |
| Setting |  |  |  | < 0.001 |
| *ICU* | 28 | 4 (14.29%) | 24 (85.71%) |  |
| *Inpatient* | 892 | 15 (1.68%) | 877 (98.32%) |  |
| *Outpatient* | 4,670 | 13 (0.28%) | 4,657 (99.72%) |  |
| *ED* | 12 | 0 (0.0%) | 12 (100%) |  |
| *Day hospital* | 43 | 0 (0.0%) | 43 (100%) |  |
| Repetitions |  |  |  | 0.48 |
| *None* | 2,645 | 13 (0.49%) | 2,632 (99.51%) |  |
| *At least one* | 3,000 | 19 (0.63%) | 2,981 (99.37%) |  |

### Figure S1 Violin plot for serum folate test results in the pediatric population.

Legend: The dashed line depicts the reference value for low values of folate (≤ 2.99 ng/mL).

### Figure S2 Histogram of the timing of serum folate exams repetitions in the pediatric population.

Legend: The dashed line depicts 90 days from a first test to a repeated test.

### Figure S3: Order setting of exams with repetitions and without repetitions in the pediatric population.

Legend: ICU, intensive care unit; ED, emergency department; inpatient refers to patients admitted to hospital, but not on ED or ICUs
